# Days alive and out of hospital at 30 days and outcomes of off-pump coronary artery bypass grafting

**DOI:** 10.1038/s41598-023-30321-8

**Published:** 2023-02-27

**Authors:** Ah Ran Oh, Seung-Hwa Lee, Jungchan Park, Jeong-Jin Min, Jong-Hwan Lee, Seung Yeon Yoo, Ji-Hye Kwon, Dan-Cheong Choi, Wooksung Kim, Hyun Sung Cho

**Affiliations:** 1 Department of Anesthesiology and Pain Medicine, Samsung Medical Center, Sungkyunkwan University School of Medicine, 81 Irwon-ro, Gangnam-gu, Seoul, 06351 Korea; 2 Department of Anesthesiology and Pain Medicine, Kangwon National University Hospital, Chuncheon, Korea; 3 Wiltse Memorial Hospital, Suwon, Korea; 4 Department of Biomedical Engineering, Seoul National University College of Medicine, Seoul, Korea; 5 Department of Biomedical Sciences, Ajou University Graduate School of Medicine, Suwon, Korea; 6 Department of Thoracic and Cardiovascular Surgery, Samsung Medical Center, Sungkyunkwan University School of Medicine, Seoul, Korea

**Keywords:** Interventional cardiology, Risk factors, Disability, Biomarkers

## Abstract

Days alive and out of hospital (DAOH) is a simple estimator based on the number of days not in hospital within a defined period. In cases of mortality within the period, DAOH is regarded as zero. It has not been validated solely in off-pump coronary artery bypass grafting (OPCAB). This study aimed to demonstrate a correlation between DAOH and outcome of OPCAB. We identified 2211 OPCAB performed from January 2010 to August 2016. We calculated DAOH at 30 and 60 days. We generated a receiver-operating curve and compared outcomes. The median duration of hospital stay after OPCAB was 6 days. The median DAOH values at 30 and 60 days were 24 and 54 days. The estimated thresholds for 3-year mortality for DAOH at 30 and 60 days were 20 and 50 days. Three-year mortality was higher for short DAOH (1.2% vs. 5.7% and 1.1% vs. 5.6% DAOH at 30 and 60 days). After adjustment, the short DAOH 30 group showed significantly higher mortality during 3-year follow-up (hazard ratio 3.07; 95% confidence interval 1.45–6.52; p = 0.004). DAOH at 30 days after OPCAB showed a correlation with 3-year outcomes. DAOH 30 might be a reliable long-term outcome measure that can be obtained within 30 days after surgery.

## Introduction

Days alive and out of hospital (DAOH) is a recent concept of outcome measure that can be easily calculated based on readily available variables^1^. It was first introduced to estimate outcomes of patients with chronic disease^2^ and has been further verified in acute disease^3^ and various surgical procedures^1,4,5^. In this regard, the Standardised Endpoints in Perioperative Medicine (StEP) initiative recommended DAOH after surgery as a reliable outcome measure in the general surgical population^6^, and recent investigations on cardiac procedures have adopted DAOH as the primary study outcome^7,8^.

The strength of DAOH is that it can be calculated simply by subtracting total days of initial or subsequent in-hospital stay from the total length of period. However, determining an adequate duration of follow-up period for DAOH is a considerable issue. It is predictable that the correlation with outcome would improve for a longer follow-up period, but DAOH can be used for more patients if its affect can be determined within a shorter period. The duration of follow-up needed to reflect outcome can vary according to surgical procedure, despite the recommended 30 days of follow-up for the general surgical population defined by the StEP initiatives^6^. In fact, DAOH of a longer period has previously been validated for high-risk surgical procedures that usually require a longer duration of in-hospital treatment^4,9^. Recent studies on cardiac surgery also analyzed DAOH at 90 or 365 days as primary outcomes^7,8^. On the other hand, off-pump coronary bypass grafting (OPCAB) lacks intraoperative cardiopulmonary bypass and was reported to show a lower rate of early complication compared with conventional coronary bypass grafting^10^. We used data of consecutive patients who underwent OPCAB in a single center where more than 1200 cases of cardiac surgery are annually performed and evaluated the associations of DAOH at 30 and 60 days with outcomes. This study aimed to evaluate whether DAOH at 30 days after OPCAB could be used to measure long-term outcome.

## Results

From January 2010 to August 2016, a total of 2211 adult patients underwent OPCAB in our institution. The results of the descriptive analysis of these patients are summarized in Table 1. The median duration of postoperative hospital stay was six (interquartile 5–8) days, and the median duration of follow-up was 1269 (interquartile 190–2674) days. The median DAOH values of survivors at 30 and 60 days were 24 (22–25) and 54 (51–55) days, respectively. The incidence of mortality was 0.4% (8/2211) for both 30- and 60-day follow-ups, while readmission rate was 5.1% (113/2211) for 30-day follow-up and 9.0% (198/2211) for 60-day follow-up (Table 1). All patient who survived at 30 days were also survivors at 60 days, so the increased number of patients in short DAOH 60 were due to re-admission during the second 30-day time interval. Of the 85 patients who were readmitted from 30 to 60 days after surgery, 54 (63.5%) patients re-admitted owing to cardiac cause.

**Table 1:** Baseline characteristics and outcomes of the entire population (N = 2211).

	Incidence or mean (± SD)
Age, years	63.2 (± 10.1)
Male	1727 (78.1)
Smoking	586 (26.5)
Body mass index	24.6 (± 3.0)
Hypertension	1755 (79.4)
Diabetes	991 (44.8)
Old myocardial infarction	198 (9.2)
Acute myocardial infarction	261 (11.8)
Ejection fraction	56.8 (± 12.3)
Previous coronary intervention	
Percutaneous intervention	387 (17.5)
Bypass grafting	9 (0.4)
Previous disease	
Peripheral arterial occlusive disease	116 (5.2)
Chronic obstructive pulmonary disease	29 (1.3)
Stroke	280 (12.7)
Chronic kidney disease	126 (5.7)
Dialysis	63 (2.8)
Heart failure	30 (1.4)
Valvular disease	12 (0.5)
Aortic disease	16 (0.7)
Drug use	
Statin	1128 (51.0)
Antiplatelet	2058 (93.1)
Renin–angiotensin–aldosterone system inhibitor	700 (31.7)
Beta blocker	724 (32.7)
Calcium channel blocker	680 (30.8)
Blood laboratory test	
Platelet, K/mcL	215.1 (± 60.1)
Albumin, g/dL	4.2 (± 0.4)
Hemoglobin, g/dL	13.2 (± 1.9)
Number of grafts	4.0 (± 1.3)
Operative variables	
Urgency operation	58 (2.6)
Operative duration, minutes	268.8 (± 69.0)
Red blood cell transfusion, pack	2.2 (± 1.6)
Postoperative course	
Acute kidney injury, any	276 (12.5)
Stage 1	181 (8.2)
Stage 2	50 (2.3)
Stage 3	45 (2.0)
30-day readmission	113 (5.1)
60-day readmission	198 (9.0)
30-day mortality	8 (0.4)
60-day mortality	8 (0.4)

Values are n (%) or mean (± SD).

**Figure 1:**
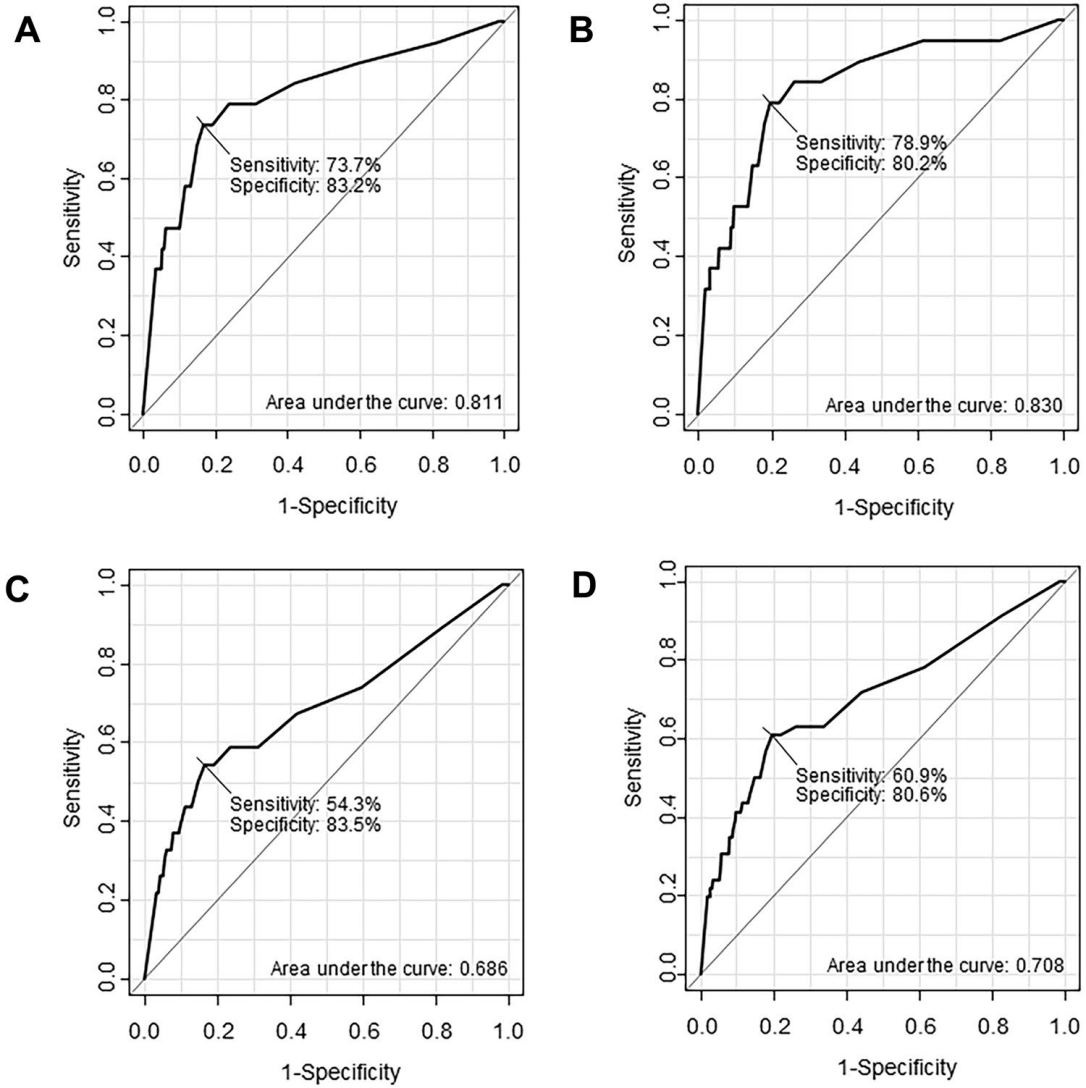
Receiver operating curves showing association between (**A**) days alive and out of hospital (DAOH) 30 and 1-year mortality, (**B**) DAOH 60 and 1-year mortality, (**C**) DAOH 30 and 3-year mortality, and (**D**) DAOH 60 and 3-year mortality.

The ROCs for the association between DAOH and mortality after OPCAB are demonstrated in Fig. 1. The area under the ROC curve for the association with 1-year mortality was 0.811 for DAOH at 30 days and 0.830 for DAOH at 60 days (Fig. 1). For 3-year mortality, this area was 0.686 for DAOH at 30 days and 0.708 for DAOH at 60 days. The thresholds associated with mortality during 3-year follow-up for DAOH at 30 and 60 days were estimated to be 20 and 50 days, respectively. The sensitivities and specificities of these thresholds are also presented in Fig. 1.

Patients who survived the first 30 or 60 days (N = 2203 for both) were divided into short and long DAOH 30 or 60 groups according to estimated threshold of DAOH for 3-year mortality (20 days for DAOH 30 and 50 days for DAOH 60). The baseline characteristics and incidences of adverse outcomes between two groups are summarized according to estimated threshold in Table 2. Between the long and short DAOH groups according to estimated threshold, the mortalities were higher in the short DAOH groups for both 30 and 60 days (1.2% vs. 5.7% for DAOH 30 and 3-year mortality, 1.1% vs. 5.6% for DAOH 60 and 3-year mortality, 0.3% vs. 3.2% for DAOH 30 and 1-year mortality, and 0.2% vs. 3.0% for DAOH 60 and 1-year mortality). This trend persisted for MACCE (7.1% vs. 14.4% for DAOH 30 and 3-year MACCE, 6.9% vs. 14.4% for DAOH 60 and 3-year MACCE, 4.5% vs. 10.1% for DAOH 30 and 1-year MACCE, and 4.3% vs. 10.0% for DAOH 60 and 1-year MACCE) and also for each composite factor of MACCE (Table 2).

**Table 2:** Baseline characteristics and clinical outcomes of survivors at postoperative 30 and 60 days according to estimated cut-off points of DAOH 30 and 60 days.

	DAOH 30 (N = 2203)			DAOH 60 (N = 2203)		
	Long > 20 (N = 1766)	Short ≤ 20 (N = 437)	P value	Long > 50 (N = 1702)	Short ≤ 50 (N = 501)	P value
Age, years	62.6 (± 10.1)	66.0 (± 9.4)	< 0.001	62.5 (± 10.1)	65.8 (± 9.3)	< 0.001
Male	1375 (77.9)	345 (78.9)	0.67	1321 (77.6)	399 (79.6)	0.37
Smoking	443 (25.1)	141 (32.3)	0.003	428 (25.1)	156 (31.1)	0.01
Body mass index	24.7 (± 3.0)	24.6 (± 3.2)	0.80	24.7 (± 3.0)	24.6 (± 3.1)	0.94
Hypertension	1384 (78.4)	363 (83.1)	0.04	1335 (78.4)	412 (82.2)	0.08
Diabetes	754 (42.7)	233 (53.3)	< 0.001	725 (42.6)	262 (52.3)	< 0.001
Old myocardial infarction	143 (8.1)	55 (12.6)	0.004	134 (7.9)	64 (12.8)	0.001
Acute myocardial infarction	193 (10.9)	66 (15.1)	0.02	183 (10.8)	76 (15.2)	0.01
Ejection fraction	58.0 (± 11.4)	51.9 (± 14.4)	< 0.001	58.0 (± 11.5)	52.9 (± 14.1)	< 0.001
Previous coronary intervention						
Percutaneous intervention	310 (17.6)	77 (17.6)	> 0.99	299 (17.6)	88 (17.6)	> 0.99
Bypass grafting	8 (0.5)	1 (0.2)	0.81	5 (0.3)	4 (0.8)	0.25
Previous disease						
Peripheral arterial occlusive disease	74 (4.2)	41 (9.4)	< 0.001	65 (3.8)	50 (10.0)	< 0.001
Chronic obstructive pulmonary disease	17 (1.0)	12 (2.7)	0.01	15 (0.9)	14 (2.8)	0.002
Stroke	183 (10.4)	95 (21.7)	< 0.001	172 (10.1)	106 (21.2)	< 0.001
Chronic kidney disease	73 (4.1)	52 (11.9)	< 0.001	71 (4.2)	54 (10.8)	< 0.001
Dialysis	34 (1.9)	28 (6.4)	< 0.001	33 (1.9)	29 (5.8)	< 0.001
Heart failure	13 (0.7)	17 (3.9)	< 0.001	13 (0.8)	17 (3.4)	< 0.001
Valvular disease	9 (0.5)	3 (0.7)	0.93	8 (0.5)	4 (0.8)	0.59
Aortic disease	11 (0.6)	5 (1.1)	0.40	8 (0.5)	8 (1.6)	0.02
Drug use						
Statin	897 (50.8)	227 (51.9)	0.71	861 (50.6)	263 (52.5)	0.48
Antiplatelet	1639 (92.8)	411 (94.1)	0.42	1582 (92.9)	468 (93.4)	0.80
Renin–angiotensin–aldosterone system inhibitor	530 (30.0)	166 (38.0)	0.002	507 (29.8)	189 (37.7)	0.001
Beta blocker	573 (32.4)	149 (34.1)	0.55	558 (32.8)	164 (32.7)	> 0.99
Calcium channel blocker	536 (30.4)	141 (32.3)	0.47	519 (30.5)	158 (31.5)	0.70
Blood laboratory test						
Platelet, K/mcL	215.7 (± 57.3)	212.7 (± 70.1)	0.36	215.7 (± 57.3)	212.9 (± 68.5)	0.35
Albumin, g/dL	4.2 (± 0.4)	4.0 (± 0.5)	< 0.001	4.2 (± 0.4)	4.0 (± 0.5)	< 0.001
Hemoglobin, g/dL	13.3 (± 1.8)	12.6 (± 2.0)	< 0.001	13.3 (± 1.8)	12.6 (± 2.0)	< 0.001
Operative variables						
Urgency operation	43 (2.4)	15 (3.4)	0.32	41 (2.4)	17 (3.4)	0.29
Operative duration, minutes	263.4 (± 65.6)	290.3 (± 77.7)	< 0.001	263.6 (± 65.7)	286.3 (± 76.6)	< 0.001
Red blood cell transfusion, pack	2.1 (± 1.5)	2.5 (± 1.7)	< 0.001	2.1 (± 1.5)	2.5 (± 1.7)	< 0.001
Number of grafts	4.1 (± 1.3)	4.2 (± 1.2)	< 0.001	4.0 (± 1.3)	4.2 (± 1.2)	0.03
Postoperative acute kidney injury	197 (11.2)	75 (17.2)	0.001	189 (11.1)	83 (16.6)	0.002
Stage 1	123 (7.0)	56 (12.8)		118 (6.9)	61 (12.2)	
Stage 2	39 (2.2)	9 (2.1)		36 (2.1)	12 (2.4)	
Stage 3	35 (2.0)	10 (2.3)		35 (2.1)	10 (2.0)	
Clinical outcome						
Three-year follow-up						
All-cause death	21 (1.2)	25 (5.7)	< 0.001	18 (1.1)	28 (5.6)	< 0.001
Graft failure	33 (1.9)	15(3.4)	0.07	31 (1.8)	17 (3.4)	0.05
Myocardial infarction	13 (0.7)	10 (2.3)	0.01	12 (0.7)	11 (2.2)	0.01
Coronary revascularization	37 (2.1)	10 (2.3)	0.95	36 (2.1)	11 (2.1)	> 0.99
Stroke	41 (2.3)	15 (3.4)	0.25	37 (2.2)	19 (3.8)	0.06
Major adverse cardio and cerebrovascular events	126 (7.1)	63 (14.4)	< 0.001	117 (6.9)	72 (14.4)	< 0.001
One-year follow-up						
All-cause death	5 (0.3)	14 (3.2)	< 0.001	4 (0.2)	15 (3.0)	< 0.001
Graft failure	21 (1.2)	13 (3.0)	0.01	19 (1.1)	15 (3.0)	0.01
Myocardial infarction	11 (0.6)	9 (2.1)	0.01	10 (0.6)	10 (2.0)	0.01
Coronary revascularization	19 (1.1)	8 (1.8)	0.30	18 (1.1)	9 (1.8)	0.28
Stroke	30 (1.7)	10 (2.3)	0.53	27 (1.6)	13 (2.6)	0.20
Major cardio and cerebrovascular events	79 (4.5)	44 (10.1)	< 0.001	73 (4.3)	50 (10.0)	< 0.001
Five-year follow-up						
All-cause death	37 (2.1)	34 (7.8)	< 0.001	33 (1.9)	38 (7.6)	< 0.001
Graft failure	38 (2.2)	18 (4.1)	0.03	36 (2.1)	20 (4.0)	0.03
Myocardial infarction	14 (0.8)	10 (2.3)	0.12	13 (0.8)	11 (2.2)	0.01
Coronary revascularization	53 (3.0)	11 (2.5)	0.70	52 (3.1)	12 (2.4)	0.53
Stroke	57 (3.2)	20 (4.6)	0.22	53 (3.1)	24 (4.8)	0.10
Major cardio and cerebrovascular events	174 (9.9)	77 (17.6)	< 0.001	164 (9.6)	87 (17.4)	< 0.001

Values are n (%) or mean (± SD).

*DAOH* days alive and out of hospital.

**Figure 2:**
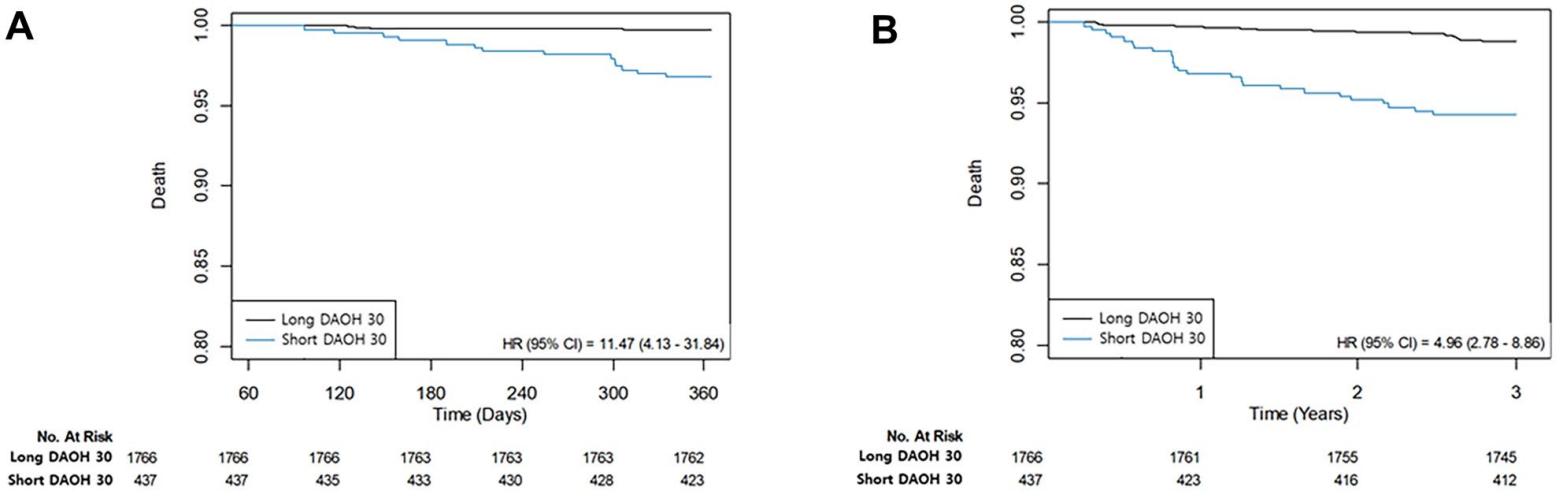
Kaplan–Meier curves for (**A**) 1-year mortality and (**B**) 3-year mortality in the groups divided by DAOH 30.

Between the short and long groups divided by DAOH 30, an IPW adjustment was conducted to improve the balance of relevant variables (Supplementary Table S1). The risk of adverse outcomes was consistently higher for the short group (HR 3.07; 95% CI 1.45–6.52; p = 0.004 for mortality and HR 1.96; 95% CI 1.34–2.87; p < 0.001 for MACCE during 3-year follow-up and HR 5.92; 95% CI 2.04–17.19; p = 0.001 for mortality and HR 2.37; 95% CI 1.50–3.73; p < 0.001 for MACCE during 1-year follow-up and HR 2.66; 95% CI 1.48–4.76; p = 0.001 for mortality and HR 1.78; 95% CI 1.27–2.48; p = 0.001 for MACCE during 5-year follow-up) (Table 3). Kaplan–Meier curves are presented in Fig. 2. For sensitivity analysis, we stratified the patients according to the different thresholds of DAOH 30 which were 15 and 18 days (Supplementary Table S2). In the subgroup analysis, there was no significant interaction with hypertension, diabetes, age over 65 years old, ejection fraction of 50%, chronic kidney disease, stroke, or acute myocardial infarction (Table 4).

**Table 3:** Risk of adverse events of survivors according to the estimated cut-off points of DAOH 30.

	Unadjusted HR (95% CI)	P value	Adjusted HR (95% CI)	P value	IPW adjusted HR (95% CI)	P value
Three-year mortality	4.96 (2.78–8.86)	< 0.001	2.93 (1.50–5.74)	0.002	3.07 (1.45–6.52)	0.004
Major adverse cardio and cerebrovascular events	2.11 (1.56–2.85)	< 0.001	1.78 (1.27–2.51)	< 0.001	1.96 (1.34–2.87)	< 0.001
One-year mortality	11.47 (4.13–31.84)	< 0.001	6.59 (2.08–20.87)	0.001	5.92 (2.04–17.19)	0.001
Major adverse cardio and cerebrovascular events	2.30 (1.59–3.33)	< 0.001	1.91 (1.26–2.90)	0.002	2.37 (1.50–3.73)	< 0.001
Five-year mortality	3.86 (2.42–6.15)	< 0.001	2.68 (1.58–4.54)	< 0.001	2.66 (1.48–4.76)	0.001
Major adverse cardio and cerebrovascular events	1.86 (1.44–2.47)	< 0.001	1.63 (1.21–2.21)	0.001	1.78 (1.27–2.48)	0.001

*HR* hazard ratio, *CI* confidence interval, *IPW* inverse probability of weighting.

**Table 4:** A subgroup analysis for the association between 3-year mortality and DAOH 30.

	Long > 20 (N = 1766)	Short ≤ 20 (N = 437)	Adjusted HR (95% CI)	P value	P for interaction
No hypertension	382	74	6.32 (1.78–22.41)	0.004	0.79
Hypertension	1384 (78.4)	363 (83.1)	5.25 (2.688–10.25)	< 0.001	
No diabetes	1012	204	5.77 (2.65–12.57)	< 0.001	0.87
Diabetes	754 (42.7)	233 (53.3)	5.26 (2.10–13.18)	< 0.001	
Age under 65 year old	961 (54.4)	167 (38.2)	3.62 (0.81–16.18)	0.09	0.75
Age over 65 years old	805 (45.6)	270 (61.8)	4.67 (2.43–8.99)	< 0.001	
Ejection fraction under 50%	1438 (81.4)	267 (61.1)	7.20 (2.03–25.53)	0.002	0.56
Ejection fraction over 50%	328 (18.6)	170 (38.9)	4.74 (2.35–9.59)	< 0.001	
No chronic kidney disease	1012 (57.3)	204 (46.7)	4.91 (2.56–9.40)	< 0.001	0.99
Chronic kidney disease	754 (42.7)	233 (53.3)	4.88 (1.01–23.51)	0.05	
No stroke	1583 (89.6)	342 (78.2)	5.02 (2.55–9.89)	< 0.001	0.99
Stroke	183 (10.4)	95 (21.7)	5.12 (1.39–18.91)	0.01	
No acute myocardial infarction	1573 (89.1)	371 (84.9)	5.76 (3.08–10.79)	< 0.001	0.65
Acute myocardial infarction	193 (10.9)	66 (15.1)	3.65 (0.61–21.87)	0.16	

**Figure 3:**
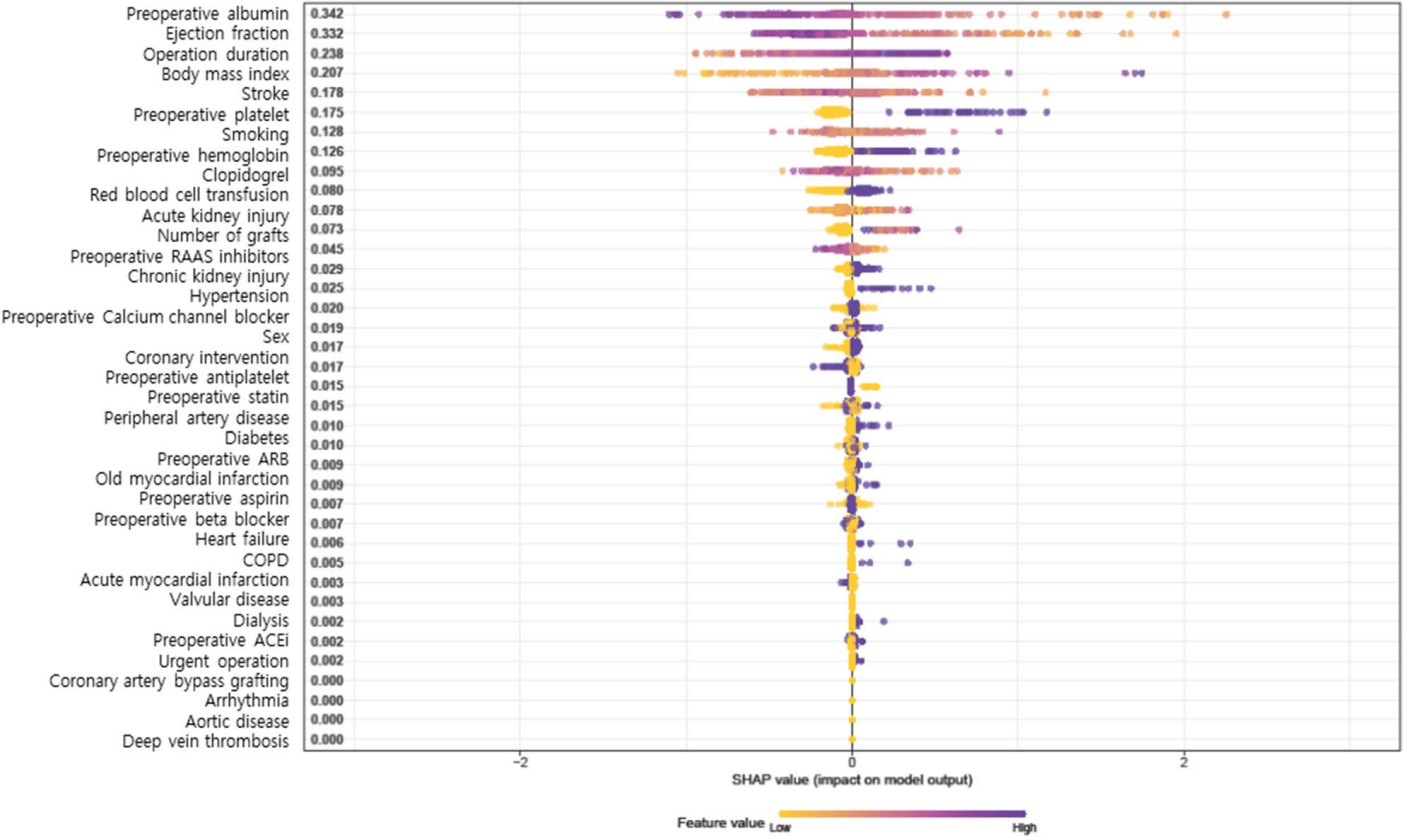
SHapley additive exPlanations (SHAP) summary plot representing the results of the extreme gradient boosting (XGB) algorithm of machine learning techniques.

The SHAP summary plot demonstrated the effects of each variable on shortening DAOH at 30 day in descending order. The most impactful variables were preoperative albumin, left ventricle ejection fraction, age, and operation duration (Fig. 3).

## Discussion

In this study, we showed that DAOH correlated well with postoperative outcome of OPCAB in long-term follow-up. DAOH on postoperative day 30 reflected mortality and MACCE during 3-year follow-up as adequately as DAOH at 60 days after OPCAB.

DAOH is an outcome measure that includes death and days out of hospital collectively. The strength of DAOH as an outcome measure is that it counts all hospitalized days and is most reflective of the patient experience as opposed to only capturing end points. Additionally, it incorporates length of stay in the metric and weighs timing of mortality, providing an indirect measure of event severity. In a perioperative setting, DAOH has additional clinical significance as an outcome measure since it does not require event adjudication. Specifically in cardiovascular research, a composite event such as MACCE has been commonly used as a study endpoint^11,12^, but these traditional time-to-composite-event analyses require a large sample size and endpoint adjudication to determine whether events occurred and caused death^3^. Moreover, this method does not distinguish the relative clinical significance of each event and counts only the first occurrence of any event^13^. This conventional approach increased the cost and complexity of clinical trials and has become one of the barriers for patient-centered trials^14^. On the other hand, DAOH can be simply calculated using administrative data. It incorporates multiple cardiovascular events into a continuous measure, resulting in reduced sample size for clinical trial. DAOH has the potential to become a promising study endpoint, but limitation remains on test duration.

In fact, DAOH has recently been recommended as a pragmatic outcome measure, with a follow-up period of 30 days in various clinical settings^1,4,5,15,16^. However, a longer period of follow-up duration is required for high-risk surgical procedures owing to longer duration of in-hospital treatment^4,9^, and DAOH of any follow-up period has not been verified in a pure cohort of OPCAB. It has been reported that OPCAB has benefits in short-term events such as transfusion, hospital stay, and stroke during 30-day follow-up^17,18^. In the present study, we evaluated the associations between long-term outcomes of OPCAB and DAOH at both 30 and 60 days follow-up and demonstrated comparable results. Additionally, we presented that the short DAOH at 30 days was associated with an increased risk of 3-year MACCE after statistical adjustments, suggesting that DAOH could be used as an alternative endpoint to circumvent the limitations of traditional composite endpoints.

We also investigated which variables are associated with shortened DAOH. The SHAP summary plot suggested that top variables affecting short DAOH at 30 days were preoperative hypoalbuminemia, low ejection fraction, and older age. These variables are consistent with the well-established risk factors associated with adverse long-term outcomes after OPCAB^19–22^. The consistency between variables affecting short DAOH and known risk factors of long-term outcome also supports that DAOH reflects outcome. However, whether modification of these variables could improve DAOH remains unclear and is beyond the scope of this study.

This study has following limitations that should be acknowledged. First, it is a single-center retrospective study, so unmeasured confounding factors might have affected our results. Also, the differences in perioperative management and surgeon experience for OPCAB might have influenced DAOH. Therefore, our results cannot be generalized to other patients, especially for the estimated thresholds of DAOH. Second, DAOH considers both cardiovascular and non-cardiovascular events. It is not necessarily expected that non-cardiovascular events will induce cardiovascular events and deaths, so incorporation of these events into the DAOH can overestimate the association between short DAOH and outcomes. Third, the diagnosis of MACCE from other clinics after discharge may not be detected due to the retrospective design of this study. So, the overall incidence of MACCE might have been underestimated. Therefore, further prospective study is required to validate the association between DAOH and MACCE after OPCAB.

Despite these limitations, this is the first study to demonstrate the correlation between DAOH at 30 days and postoperative long-term outcomes in OPCAB. DAOH combines both the length of time a patient spends in the hospital with their overall well-being outside of the hospital, which may provide a more comprehensive and accurate prediction of outcomes. By observing during a short period with a simple and straightforward metric, the data are rapidly obtainable and allow more flexible responses in practice, without incumbent delays of long follow-up periods. To reflect these outcomes clinically, clinicians can use DAOH at 30 days as a routine post-operative measure to monitor and assess patient progress after OPCAB.

## Conclusion

DAOH is a simple measure that is readily available using existing data sources. In OPCAB, DAOH at 30 days might be a valid outcome tool for predicting long-term outcomes. Further studies are required to establish the consensus support for the use of DAOH in OPCAB.

## Methods

### Study population, data collection, and study endpoints

This study is a retrospective observational cohort study and was approved by the Institutional Review Board at Samsung Medical Center (2022-05-087). It was conducted according to the Declaration of Helsinki, and the report followed the Strengthening the Reporting of Observational Studies in Epidemiology (STROBE) guidelines. Written informed consent from individual patients was waived by the Institutional Review Board at Samsung Medical Center/ethics committee (2022-05-087) considering the minimal risk for the participants and retrospective nature of the study (Institutional Review Board at Samsung Medical Center/Sungkyunkwan University).

We reviewed the data of consecutive adult patents who underwent OPCAB at our institution between January 2010 and August 2016. In patients who underwent re-operation, only the first operation was included for analysis. An independent investigator who was not otherwise involved in this study organized clinical, laboratory, and outcome data. The mortality data in our electronic hospital record system are based on National Population Registry of Korea, so death outside the institution was also considered in this study.

In our institution, all patients who required coronary bypass grafting were preferentially indicated for OPCAB. However, on-pump coronary bypass grafting was performed in cases of severe hemodynamic instability, impaired left ventricular function, recent myocardial infarction, and whenever accuracy or patency of the distal anastomosis was in doubt.

The primary endpoint of this study was to evaluate the association between mortality during 3-year follow-up and DAOH at 30 and 60 days. For secondary endpoints, mortality during 1- and 5-year follow-ups and major cardiovascular and cerebrovascular events (MACCE) during 1-, 3-, and 5-year follow-up were evaluated. MACCE was defined as a composite of all-cause death, graft failure, coronary revascularization, myocardial infarction, and stroke^23^.

### Calculation of DAOH

Calculation of DAOH is described in a previous study^1^. The number of days spent out of hospital was obtained by subtracting total duration of initial or subsequent in-hospital days from the total defined length of period (30 or 60 days). As DAOH was 0 in patients who died within the defined period, DAOH ranged from 0 to the defined length of period, and a smaller number indicates a worse outcome. In order to evaluate the association between DAOH and long-term outcome, we excluded the patients who died within 30 or 60 days and enrolled only survivors after 30 or 60 days, respectively.

### Statistical analysis

We presented descriptive data of the entire population and the association with outcomes among survivors of the defined periods of DAOH. In the descriptive analysis, continuous data were presented as mean ± standard deviation, and categorical variables were presented as number with incidence. We constructed receiver operating curve (ROC) plots to estimate thresholds of DAOH at each follow-up day. The best thresholds correspond to the maximum Youden index, which represents the balance between sensitivity and specificity that maximizes the overall accuracy of the classifier. The survivors were divided according to these thresholds, and the incidence of adverse outcomes between the groups were compared. We constructed Kaplan–Meier curves and compared groups with log-rank test. For DAOH of 30 days, we compared the risk of adverse outcomes between the groups using inverse probability of weighting (IPW) and provided it as hazard ratio (HR) with 95% confidence interval (CI). We also investigated the effect of each variable on short DAOH 30 using a machine learning technique with an extreme gradient boosting (XGB) algorithm provided by the xgboost package of R. The results were presented as a SHapley Additive exPlanations (SHAP) summary plot, which illustrates the intensity and direction of impact on the outcome of interest. The SHAP value is determined by comparing the prediction of the model with and without each variable^24^. All statistical analyses were performed with R 4.1 (Vienna, Austria; http://www.R-project.org/). All tests were two-tailed, and p < 0.05 was considered statistically significant.

### Ethical approval

This study was approved by the Institutional Review Board at Samsung Medical Center. Reference number: 2022-05-087.

## Supplementary Information

Supplementary Information.

## Data Availability

The individual deidentified participant data will be shared as all analyzable dataset related to the study will be available. Study protocol and statistical analysis plan will be available. These will be shared on a request basis, immediately following publication, ending 10 years after the publication. The data will be shared on a request basis for anyone to validate our findings. The data will be shared as csv file via email. Please directly contact the corresponding author to request data sharing.

## Abbreviations

DAOH: Days alive and out of hospital
OPCAB: Off-pump coronary artery bypass grafting
