# Supplementary material for: Days alive and out of hospital at 30 days and outcomes of off-pump coronary artery bypass grafting
Source: Sci Rep. 2023 Feb 27;13:3359. doi: 10.1038/s41598-023-30321-8 (PMC9971038; doi:10.1038/s41598-023-30321-8)
Supplement: Supplementary file 1 — Supplementary Information. [file 41598_2023_30321_MOESM1_ESM.docx]

**Supplementary Table 1. Improved Balance of Variables After IPW Adjustment**

|  | **ASD before IPW** | **ASD after IPW** |
| --- | --- | --- |
| Age, years | 34.9 | 4.4 |
| Male | 2.6 | 2.7 |
| Smoking | 15.9 | 3.8 |
| Body mass index | 1.3 | 1 |
| Hypertension | 11.9 | 5.6 |
| Diabetes | 21.4 | 2.9 |
| Old myocardial infarction | 14.8 | 2.8 |
| Acute myocardial infarction | 12.4 | 1.4 |
| Ejection fraction | 47.1 | 0.1 |
| **Previous coronary intervention** |  |  |
| Percutaneous intervention | 0.2 | 52 |
| Bypass grafting | 3.8 | 4.6 |
| **Previous disease** |  |  |
| Peripheral arterial occlusive disease | 20.8 | <0.1 |
| Chronic obstructive pulmonary disease | 13.2 | 0.4 |
| Stroke | 31.4 | 0.1 |
| Chronic kidney disease | 28.9 | 5.2 |
| Dialysis | 22.6 | 0.3 |
| Heart failure | 21.1 | 6.8 |
| Valvular disease | 2.3 | 3.7 |
| Aortic disease | 5.6 | 2.4 |
| **Drug use** |  |  |
| Statin | 2.3 | 2.8 |
| Antiplatelet | 5 | 0.2 |
| Renin-angiotensin-aldosterone system inhibitor | 16.9 | 3.8 |
| Beta blocker | 3.5 | 0.4 |
| Calcium channel blocker | 4.1 | 2.2 |
| Blood laboratory test |  |  |
| Platelet, K/mcL | 4.6 | 3.5 |
| Albumin, g/dL | 59.7 | 1.8 |
| Hemoglobin, g/dL | 39.1 | 2.2 |
| Operative variables |  |  |
| Urgency operation | 5.9 | 2.7 |
| Operative duration, minutes | 37.3 | 3.7 |
| Red blood cell transfusion, pack | 23.8 | 0.6 |

ASD, absolute standardized difference; IPW, inverse probability of weighting.

**Supplementary Table 2. Baseline characteristics and clinical outcomes of survivors at postoperative 30 days according to different cut-off points of DAOH 30 (15 and 18).**

|  | Long > 15 | Short ≤ 15 | P-value | Long > 18 | Short ≤ 18 | P-value |
| --- | --- | --- | --- | --- | --- | --- |
|  | (N=1970) | (N=233) |  | (N=1863) | (N=340) |  |
| Age, years | 62.8 (±10.1) | 66.6 (±9.4) | <0.001 | 1444 (77.5) | 276 (81.2) | 0.15 |
| Male | 1534 (77.9) | 186 (79.8) | 0.55 | 466 (25.0) | 118 (34.7) | <0.001 |
| Smoking | 499 (25.3) | 85 (36.5) | <0.001 | 466 (25.0) | 118 (34.7) | <0.001 |
| Body mass index | 24.7 (±3.0) | 24.6 (±3.2) | 0.9 | 24.7 (±3.0) | 24.6 (±3.1) | 0.62 |
| Hypertension | 1549 (78.6) | 198 (85.0) | 0.03 | 1463 (78.5) | 284 (83.5) | 0.04 |
| Diabetes | 858 (43.6) | 129 (55.4) | 0.001 | 810 (43.5) | 177 (52.1) | 0.004 |
| Old myocardial infarction | 174 (8.8) | 24 (10.3) | 0.54 | 158 (8.5) | 40 (11.8) | 0.07 |
| Acute myocardial infarction | 221 (11.2) | 38 (16.3) | 0.03 | 205 (11.0) | 54 (15.9) | 0.01 |
| Ejection fraction | 57.1 (±12.2) | 54.1 (±13.2) | <0.001 | 57.6 (±11.8) | 52.4 (±14.1) | <0.001 |
| **Previous coronary intervention** |  |  |  |  |  |  |
| Percutaneous intervention | 353 (17.9) | 34 (14.6) | 0.24 | 332 (17.8) | 55 (16.2) | 0.51 |
| Bypass grafting | 9 (0.5) | 0 | 0.62 | 8 (0.4) | 1 (0.3) | >0.99 |
| **Previous disease** |  |  |  |  |  |  |
| Peripheral arterial occlusive disease | 88 (4.5) | 27 (11.6) | <0.001 | 83 (4.5) | 32 (9.4) | <0.001 |
| Chronic obstructive pulmonary disease | 22 (1.1) | 7 (3.0) | 0.04 | 17 (0.9) | 12 (3.5) | <0.001 |
| Stroke | 224 (11.4) | 54 (23.2) | <0.001 | 200 (10.7) | 78 (22.9) | <0.001 |
| Chronic kidney disease | 94 (4.8) | 31 (13.3) | <0.001 | 82 (4.4) | 43 (12.6) | <0.001 |
| Dialysis | 44 (2.2) | 18 (7.7) | <0.001 | 35 (1.9) | 27 (7.9) | <0.001 |
| Heart failure | 24 (1.2) | 6 (2.6) | 0.16 | 18 (1.0) | 12 (3.5) | <0.001 |
| Valvular disease | 11 (0.6) | 1 (0.4) | >0.99 | 9 (0.5) | 3 (0.9) | 0.6 |
| Aortic disease | 12 (0.6) | 4 (1.7) | 0.14 | 11 (0.6) | 5 (1.5) | 0.16 |
| **Drug use** |  |  |  |  |  |  |
| Statin | 1016 (51.6) | 108 (46.4) | 0.15 | 953 (51.2) | 171 (50.3) | 0.82 |
| Antiplatelet | 1836 (93.2) | 214 (91.8) | 0.5 | 1730 (92.9) | 320 (94.1) | 0.47 |
| Renin-angiotensin-aldosterone system inhibitor | 603 (30.6) | 93 (39.9) | 0.01 | 562 (30.2) | 134 (39.4) | 0.001 |
| Beta blocker | 641 (32.5) | 81 (34.8) | 0.54 | 601 (32.3) | 121 (35.6) | 0.25 |
| Calcium channel blocker | 588 (29.8) | 89 (38.2) | 0.01 | 559 (30.0) | 118 (34.7) | 0.1 |
| **Blood laboratory test** |  |  |  |  |  |  |
| Platelet, K/mcL | 215.4 (±58.0) | 212.4 (±75.2) | 0.47 | 215.6 (±57.8) | 212.0 (±71.0) | 0.3 |
| Albumin, g/dL | 4.2 (±0.4) | 3.9 (±0.5) | <0.001 | 4.2 (±0.4) | 3.9 (±0.5) | <0.001 |
| Hemoglobin, g/dL | 13.3 (±1.8) | 12.3 (±2.1) | <0.001 | 13.3 (±1.8) | 12.4 (±2.0) | <0.001 |
| **Operative variables** |  |  |  |  |  |  |
| Urgency operation | 48 (2.4) | 10 (4.3) | 0.15 | 45 (2.4) | 13 (3.8) | 0.19 |
| Operative duration, minutes | 266.4 (±67.8) | 288.9 (±75.4) | <0.001 | 265.0 (±67.1) | 289.2 (±75.7) | <0.001 |
| Red blood cell transfusion, pack | 2.2 (±1.5) | 2.6 (±1.8) | <0.001 | 2.1 (±1.5) | 2.5 (±1.8) | <0.001 |
| **Postoperative acute kidney injury** | 224 (11.4) | 48 (20.6) | <0.001 | 211 (11.3) | 61 (17.9) | 0.002 |
| Stage 1 | 141 (7.2) | 38 (16.3) |  | 134 (7.2) | 45 (13.2) |  |
| Stage 2 | 42 (2.1) | 6 (2.6) |  | 40 (2.1) | 8 (2.4) |  |
| Stage 3 | 41 (2.1) | 4 (1.7) |  | 37 (2.0) | 8 (2.4) |  |
| **Clinical outcome** |  |  |  |  |  |  |
| Three-year follow-up |  |  |  |  |  |  |
| All-cause death | 28 (1.4) | 18 (7.7) | <0.001 | 23 (1.2) | 23 (6.8) | <0.001 |
| Graft failure | 41 (2.1) | 7 (3.0) | 0.5 | 36 (1.9) | 12 (3.5) | 0.1 |
| Myocardial infarction | 17 (0.9) | 6 (2.6) | 0.04 | 16 (0.9) | 7 (2.1) | 0.09 |
| Coronary revascularization | 41 (2.1) | 6 (2.6) | 0.8 | 40 (2.1) | 7 (2.1) | 1 |
| Stroke | 49 (2.5) | 7 (3.0) | 0.8 | 46 (2.5) | 10 (2.9) | 0.75 |
| Major adverse cardio and cerebrovascular events | 153 (7.8) | 36 (15.5) | <0.001 | 138 (7.4) | 51 (15.0) | <0.001 |
| One-year follow-up |  |  |  |  |  |  |
| All-cause death | 10 (0.5) | 9 (3.9) | <0.001 | 6 (0.3) | 13 (3.8) | <0.001 |
| Graft failure | 27 (1.4) | 7 (3.0) | 0.1 | 24 (1.3) | 10 (2.9) | 0.04 |
| Myocardial infarction | 15 (0.8) | 5 (2.1) | 0.08 | 14 (0.8) | 6 (1.8) | 0.13 |
| Coronary revascularization | 23 (1.2) | 4 (1.7) | 0.69 | 22 (1.2) | 5 (1.5) | 0.86 |
| Stroke | 35 (1.8) | 5 (2.1) | 0.89 | 32 (1.7) | 8 (2.4) | 0.56 |
| Major cardio and cerebrovascular events | 100 (5.1) | 23 (9.9) | 0.004 | 88 (4.7) | 35 (10.3) | <0.001 |
| Five-year follow-up |  |  |  |  |  |  |
| All-cause death | 48 (2.4) | 23 (9.9) | <0.001 | 43 (2.3) | 28 (8.2) | <0.001 |
| Graft failure | 47 (2.4) | 9 (3.9) | 0.26 | 42 (2.3) | 14 (4.1) | 0.07 |
| Myocardial infarction | 18 (0.9) | 6 (2.6) | 0.05 | 17 (0.96) | 7 (2.1) | 0.11 |
| Coronary revascularization | 58 (2.9) | 6 (2.6) | 0.91 | 56 (3.0) | 8 (2.4) | 0.63 |
| Stroke | 67 (3.4) | 10 (4.3) | 0.61 | 62 (3.3) | 15 (4.4) | 0.40 |
| Major cardio and cerebrovascular events | 208 (10.6) | 43 (18.5) | 0.001 | 190 (10.2) | 61 (17.9) | <0.001 |
